# Fixation failure and early loss of reduction with the use of suture anchors for surgical repair of acromioclavicular joint dislocation: a case series

**DOI:** 10.1016/j.jseint.2024.06.011

**Published:** 2024-07-08

**Authors:** Erel Ben-Ari, Dashaun A. Ragland, Andrew J. Cecora, Mandeep S. Virk

**Affiliations:** Division of Shoulder and Elbow Surgery, Department of Orthopedic Surgery, NYU Grossman School of Medicine, NYU Langone Orthopedic Hospital, NYU Langone Health, New York, NY, USA

**Keywords:** Acromioclavicular joint, Dislocation, Anchor, Shoulder instability, Fixation failure, Arthroscopy

## Abstract

**Background:**

Suture anchors have been used in surgical repair of acromioclavicular joint (ACJ) dislocation. While some reports indicate favorable results, others emphasize less promising outcomes. This case series reports our experience with suture anchors for surgical treatment of ACJ dislocation.

**Methods:**

Clinical and radiographic outcomes in three patients treated operatively for ACJ dislocations were reviewed. In all patients, two suture anchors were inserted in the coracoid (unicortical) and #5 nonabsorbable suture from the suture anchor was shuttled through drill holes in the clavicle and tied over two button devices. The coracoclavicular ligaments were reconstructed using a figure of eight semitendinosus allograft around the coracoid and clavicle. Postoperatively, sling immobilization was used for 6 weeks, and physical therapy was initiated at 6 weeks with contact activity allowed at 6 months.

**Results:**

Three male patients underwent treatment for Rockwood type 3 (chronic; n = 1) and type 5 (n = 2) ACJ dislocations. Loss of reduction was noted within 6 weeks postoperatively. Two patients exhibited failure due to complete suture anchor pullout and the third patient had partial pull out of one of the anchors. Additionally, the third patient also suffered a coracoid fracture adjacent to the anchor’s placement after sustaining direct trauma to his shoulder, one-year postoperatively.

**Conclusion:**

In our case series, we found a high rate of fixation failure and early loss of reduction with the use of suture anchors for the treatment of ACJ dislocation. These findings should be taken into consideration when selecting an appropriate implant for fixation of ACJ dislocation.

Acromioclavicular joint (ACJ) dislocation accounts for approximately 9% to 12% of shoulder girdle injuries.^7^^,^^12^^,^^17^ Surgical intervention is often recommended for Rockwood types 4-6 and for type 3 injuries in certain situations.^7^ Various surgical fixation techniques have been described for ACJ dislocations with anatomical reconstruction of coracoclavicular ligaments (ACCR) emerging as a popular technique in recent years.^2^^,^^7^^,^^14^^,^^17^

The ACCR technique involves reconstruction of the conoid and trapezoid ligaments with an autograft or allograft. Various types of implants are available for acromioclavicular (AC) and coracoclavicular (CC) fixation, including screws, cortical buttons, and suture anchors. Suture anchors are an attractive option for distal fixation on coracoid as they can be inserted in a minimally invasive fashion, are associated with shorter operative times and reduced risk of neurovascular injury compared to other techniques that require dissection and passage of fixation devices around the coracoid base.^2^^,^^4^^,^^7^^,^^17^ However, inconsistent data has been reported on the clinical and biomechanical outcomes following treatment of AC joint dislocation with suture anchors. Jerosch et al^11^ evaluated eight different ACJ reconstruction techniques in cadaveric shoulders and found that fixation with suture anchors placed at the base of the coracoid were most effective in restoring AC joint anatomy. Additionally, Chernchujit et al^5^ demonstrated that suture anchor fixation provides an effective stabilization for physiologic motion between the clavicle and coracoid. Conversely, in a biomechanical comparison study, Harris et al^8^ showed that suture anchors had comparable tensile strength of the native CC ligaments but less than half their stiffness, which could potentially lead to complications.

This case series presents clinical and radiographic outcomes of three consecutive patients with AC joint dislocation, in the senior author’s practice, that were treated with suture anchors for distal fixation on the coracoid and were associated with an early loss of fixation and reduction.

## Methods

This retrospective cohort study conducted at a single center received approval from the institutional review board.

In 2017, the senior author transitioned his surgical approach for ACJ reconstruction, from utilizing the cortical button system to employing suture anchors for distal fixation on the coracoid. This study includes three consecutive patients who underwent surgical repair of AC joint dislocations using suture anchor. Preoperative radiographs, surgical records, office notes and postoperative radiographs were reviewed for this study.

### Surgical technique and postoperative protocol

The surgery was performed under regional anesthesia (interscalene block) and intravenous sedation in the beach chair position. An 8 cm saber incision, starting posterior to the clavicle and extending distally to the tip of the coracoid process, was used in all cases. Full thickness subcutaneous flaps were elevated to expose the underlying deltotrapezial fascia, which was incised to expose the dorsal clavicle without elevating the deltoid or trapezius muscles. The AC joint capsule was incised dorsally and elevated off the lateral end of clavicle. The distal clavicle was excised using an oscillating saw (5 mm). Two 2 mm drill holes were drilled in superior-inferior direction through the clavicle at distances of 25 mm and 45 mm from the lateral end of the clavicle, which correspond to the anatomic location of the conoid and trapezoid ligaments on the clavicle and these drill holes will be subsequently used to shuttle sutures from the two suture anchors in the coracoid. The deltopectoral interval was identified and the cephalic vein was retracted medially. The clavipectoral fascia was incised and the coracoacromial ligament and coracoid process were identified. The medial and lateral borders of coracoid were dissected subperiosteally, and a loop suture was passed around the coracoid for shuttling the allograft. Two 5.5 mm metallic suture anchors loaded with a #5 nonabsorbable suture (Acumed Inc., Hillsboro, OR, USA) were inserted on the dorsal surface of coracoid separated by at least 5 mm bone bridge. Allograft tendon (Semitendinosus) was prepped at the back table and shuttled around the coracoid base along with a #2 nonabsorbable suture (Arthrex Inc., Naples, FL, USA). The graft limbs were crossed over the dorsum of coracoid and brought anterior and posterior to the clavicle. The sutures from the anchors were then shuttled through the drill holes. The joint was reduced and verified by fluoroscopy imaging and the sutures were tied over a button device on the dorsal clavicle. The two limbs of the graft were tied to each other and secured with nonabsorbable sutures. The ACJ capsule was repaired in pants over vest configuration using #2 nonabsorbable suture.

Postoperatively, the arm was immobilized in a sling for six weeks. During this period, only therapist-supervised passive forward elevation (<90 degrees) and passive external and internal rotation with the arm at the side, were allowed. Active range of motion, certain passive motion (internal rotation behind the back, abduction, and external rotation) and pendulum exercises were restricted. Progressive range of motion exercises began in the seventh week. Resistive exercises and strengthening exercises commenced three months postoperatively and contact sports were allowed after 6 months.

### Radiographic assessment

Preoperative, and post operative radiographs (Grashey, axillary, and scapular Y views) and intraoperative fluoroscopic images were reviewed for reduction of the AC joint, CC distance and signs of fixation failure. Postoperative radiographs were obtained postoperative visit, which occurred at 2 weeks, 6 weeks, 3 months, and at one-year mark if applicable.

## Results

### Injury mechanism

**Figure 1:**
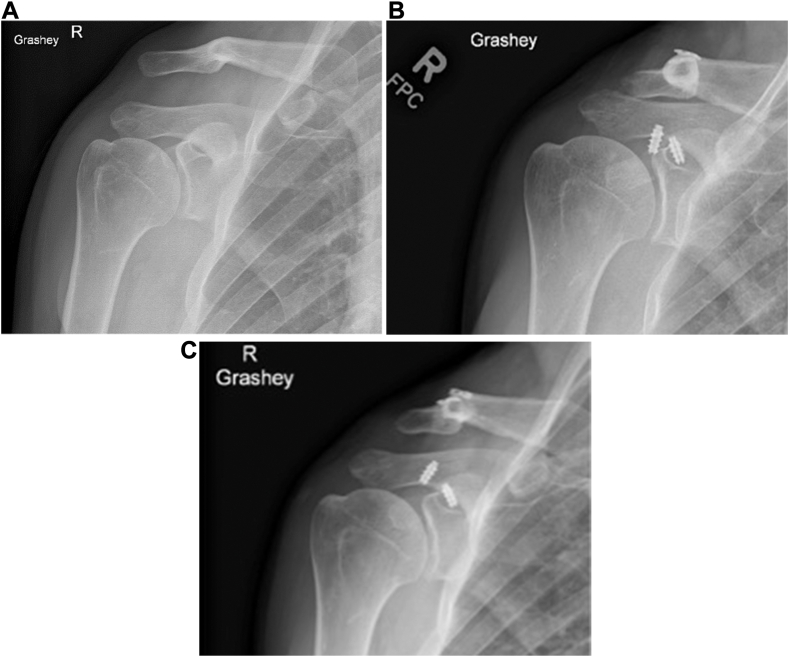
(**A**) Preoperative X-ray imaging (Grashey view) demonstrating Rockwood type 5 ACJ dislocation. (**B**) Two weeks postoperative X-ray imaging (Grashey view) demonstrating partial anchor loosening, with no loss of ACJ reduction. (**C**) Six weeks postoperative X-ray imaging (Grashey view) demonstrating anchor pullout and loss of ACJ reduction. *ACJ*, acromioclavicular joint.

**Figure 2:**
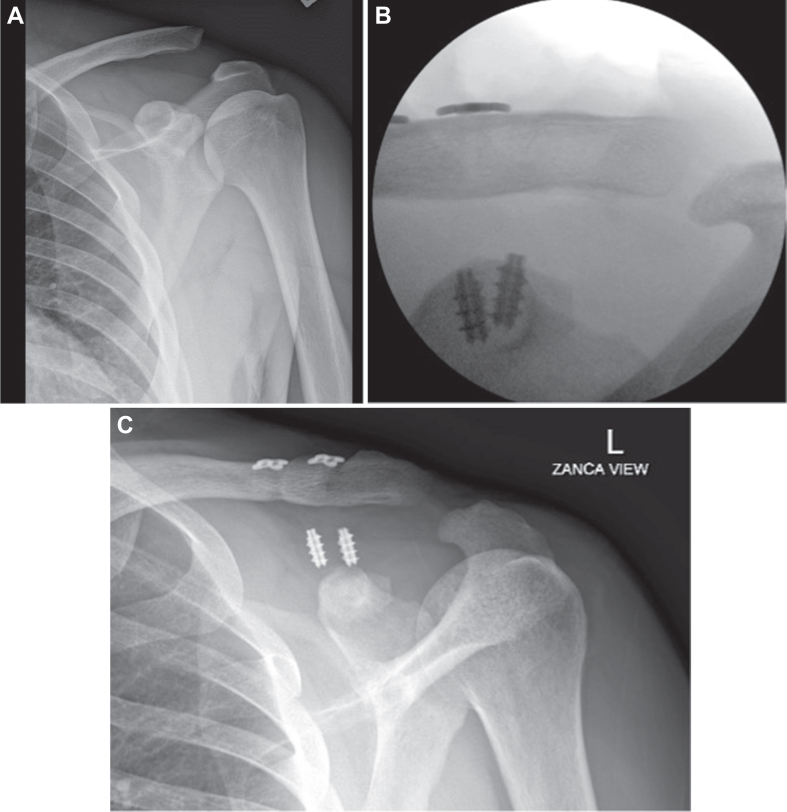
(**A**) Preoperative X-ray imaging (Zanca^20^ view) demonstrating Rockwood type 3 ACJ dislocation. (**B**) Intraoperative fluoroscopic imaging showing ACJ reduction with suture anchors fixation. (**C**) Six weeks postoperative X-ray imaging (Grashey view) demonstrating anchor pullout and loss of ACJ reduction. *ACJ*, acromioclavicular joint.

**Figure 3:**
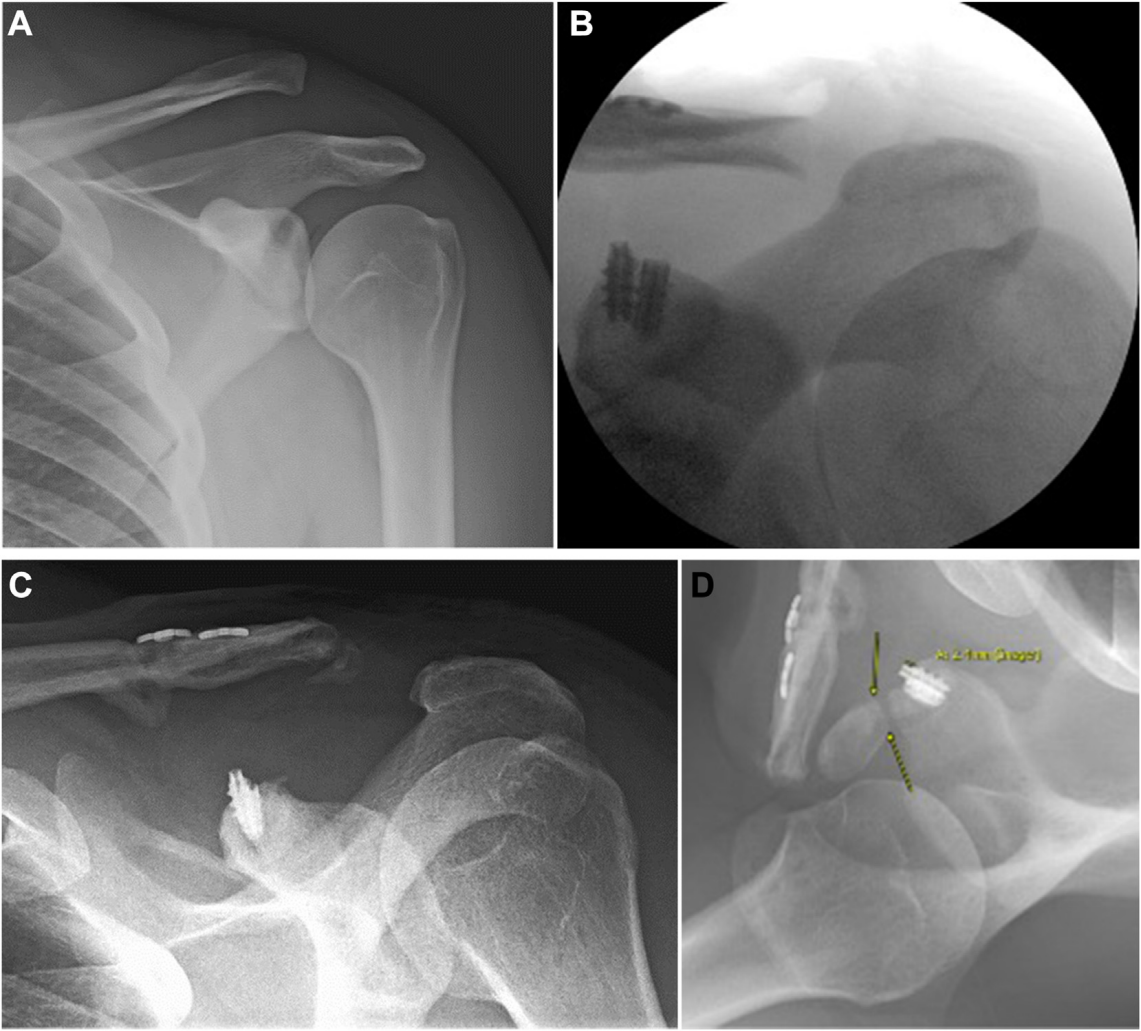
(**A**) Preoperative X-ray imaging (Zanca^20^ view) demonstrating Rockwood type 5 ACJ dislocation. (**B**) Intraoperative fluoroscopic imaging showing ACJ reduction with suture anchors fixation. (**C**) Three weeks postoperative X-ray imaging (Zanca^20^ view) demonstrating loosening of suture anchors and partial loss of reduction of the ACJ. (**D**) Two years postoperative X-ray imaging (Axillary view) demonstrating a coracoid fracture distal to the suture anchors. *ACJ*, acromioclavicular joint.

Case 1 is a 44-year-old, right-hand dominant male who sustained a right Rockwood type 5 ACJ dislocation after a fall at home (Fig. 1*A*). Three weeks after the injury, the patient underwent AC joint reconstruction as described in the methods section. The second case (Case 2) is a 30-year-old right-handed male who sustained a left Rockwood type 3 ACJ separation after a fall while snowboarding (Fig. 2*A*). The patient was initially treated nonoperatively but due to persistent pain and shoulder dysfunction the patient decided to undergo AC joint reconstruction ten months postinjury. The third case is a 22-year-old, left-handed male who sustained a left Rockwood type 5 ACJ separation after a fall from motorbike. (Fig. 3*A*) He underwent AC joint reconstruction two weeks after the injury as described in methods section (Table 1).

**Table I:** Patient characteristics.

Case #	Age (y)	Gender (male/female)	Arm dominance (right/left)	Injury side	Mechanism of injury	Rockwood type (1-6)	Time from injury to surgery (Weeks)
1	44	Male	Right	Right	Fall	5	3
2	30	Male	Right	Left	Snowboard accident	3	40
3	22	Male	Left	Left	Motorcycle accident	5	2

### Intraoperative and postoperative outcome

Intraoperatively, no complications were noted and review of intraoperative fluoroscopic imaging demonstrated anatomical reduction of the ACJ in all three patients. Case 1 reported hearing a popping sound in the shoulder while showering accompanied by minimal pain. At the two-week postoperative visit, radiographs demonstrated partial anchor dislodgement, but the ACJ joint remained reduced (Fig. 1*B*). The patient continued with the prescribed postoperative protocol. Six-week radiographs resembled those from the prior visit, but at the three-month visit complete anchor displacement and loss of ACJ reduction were evident (Fig. 1*C*). The patient had revision surgery three months after the initial AC joint reconstruction. Intraoperatively, the allograft was intact but there was complete dislodgement of the suture anchors, which were removed. Five months after the initial surgery and two months after the revision surgery, the patient developed surgical site infection and underwent repeat débridement surgery and allograft removal along with postoperative antibiotic treatment. At the last follow-up, one-year after the initial ACCR, no signs of infection were noted, and the patient regained full shoulder function with no pain. At the last follow-up one-year after the initial ACCR, no signs of infection were noted, and the patient regained full shoulder function with no pain.

Similar to Case 1, Case 2 experienced a popping sound after an abrupt shoulder movement, accompanied by severe pain four weeks after the surgery. Radiographs obtained at that time (∼6 weeks after surgery) revealed suture anchor pullout and an increased CC distance compared to prior imaging obtained at the 2-week follow-up (Fig. 2*C*). The patient was offered revision surgery but he declined because his pain was improving by the time he was seen in the office. At 3-month follow-up, the patient’s pain had considerably improved, and he had improvements in active shoulder range of motion and decided to continue with observation.

Case 3 injured his operative shoulder 3 weeks after the surgery when his arm was jerked after the car (passenger in a cab) made a sudden stop throwing him towards the front seat. Radiographs in the emergency room showed loss of ACJ reduction, partial pullout of one anchor, and an increased CC distance compared to imaging obtained at the two-week follow-up (Fig. 3*C*). Two years later, he sustained a coracoid fracture distal to the suture anchor after a car accident. (Fig. 3*D*) No further clavicle or ACJ displacement was noted on subsequent imaging, and he elected to continue nonoperative treatment.

## Discussion

This case series demonstrates our experience with the use of suture anchors for surgical fixation of ACJ dislocation. We found a high rate of fixation failure (anchor pull out) in our series during the early postoperative period resulting in loss of AC joint reduction. Furthermore, one patient experienced a coracoid fracture distal to the suture anchor.

Over the years numerous implant options and surgical techniques have been proposed for treating AC joint dislocations. However, there is currently no consensus on the ideal surgical treatment and implant type.^2^^,^^7^^,^^10^^,^^15^^,^^17^^,^^18^ The stability of the AC joint relies on the contributions of both the CC ligaments for vertical stability and the AC capsule for horizontal stability. Regardless of the implant utilized, the goal is to achieve and sustain the reduction of the CC interval and the AC joint, ensuring the ability to withstand physiological loads and restore stability for adequate biological healing of the graft.^3^^,^^10^^,^^12^^,^^15^^,^^18^

In recent years, the popularity of ACCR as a surgical technique has grown, primarily due to its biomechanical effectiveness in restoring vertical stability to the ACJ joint.^2^^,^^7^^,^^14^^,^^17^^,^^19^ Notably, Spencer et al^19^ observed the lowest rates of revision surgery and radiographic failures when employing a combined approach of anatomic CC ligaments reconstruction through coracoid and clavicular bone tunnels and nonanatomic reconstruction with allograft loop CC fixation. This approach was more effective than using each method alone. Furthermore, improved outcomes were reported in cases where both the CC and AC ligaments were addressed during surgeries.^1^^,^^12^^,^^13^^,^^16^^,^^18^^,^^21^

We found a high failure rate with use of suture anchor for surgical treatment of AC joint dislocation. This is in contrast with previous reports which confirmed the effectiveness of suture anchor fixation in the treatment of ACJ dislocations.^4^, ^5^, ^6^^,^^9^^,^^11^^,^^13^^,^^17^^,^^21^ Studies supporting the use of suture anchor for ACJ reconstruction suggest that the anchor size and number of suture strands can impact the stability of the fixation. Imhoff et al^9^ and Chernchujit et al^6^ compared mechanical properties of the native CC ligament with various reconstruction techniques. They found that a single #5 FiberWire (Arthrex Inc., Naples, FL, USA) demonstrated higher failure rates compared to the native CC ligaments. However, the use of two #5 FiberWire provided tensile strength exceeding that of the native ligaments. Chernchujit et al^5^ demonstrated that a 5 mm corkscrew loaded with #5 FibreWire placed at the base of the coracoid showed no fixation failure, in contrast to 2.8 mm suture anchors (Fastak; Arthrex Inc. Naples, FL) loaded with #2 FiberWire, which led to fixation failures. Liu et al^12^ reported similar outcomes when using either 3.5 mm or 5 mm double-loaded suture anchors (Twinfix; Smith & Nephew, Memphis, TN, USA) for CC fixation. The choice of anchor size was based on patient size, with the utilization of 5 mm anchors in larger individuals. Zhang et al^21^ utilized two 5 mm suture anchors (Smith-Nephew, USA) with four sutures attached to each anchor. In their series of 28 patients, they documented two cases of anchor pullout in obese individuals, attributing the failure to excessive active shoulder motion during the early postoperative period. In addition, to minimize anchor pullout, the authors also recommended positioning the anchors at the base of the coracoid to achieve the best bone fixation as well as avoiding a suture trajectory aligned with the long axis of the anchors.

We did not find any risk factors that would predispose these patients to risk of early failure other than physiologic loads during routine activities. However, fixation failure was observed in the early postoperative phase, and eventual loss of reduction was evident in all patients. All patients were males with good bone stock and no medical history suggestive of osteoporosis. During the postoperative period, one patient experienced trauma to the operated shoulder, resulting in a partial pullout of a single anchor. Additionally, this patient suffered a coracoid fracture; however, it occurred distal to the placement of the anchors, and the anchors themselves remained stable. Our surgical technique involved stabilization of the AC joint in both the vertical and horizontal planes. To address vertical stability, we used two suture anchors (5.5 mm outer diameter; Acumed Inc., Hillsboro, OR, USA) that were inserted unicortically in the coracoid and loaded with #5 nonabsorbable suture. The sutures were passed through the drill holes in the clavicle directed along the axis of conoid and trapezoid ligaments and tied over button devices. A semitendinosus allograft was looped around the clavicle and coracoid to provide additional biologic stability. In addition, we addressed horizontal stability by reconstructing the ACJ capsule in pants over vest configuration using #2 nonabsorbable suture. Intraoperative imaging demonstrated reduction of the CC interval and AC joint articulation and appropriate placement of the suture anchors.

The senior author has utilized these suture anchors alongside clavicle plates for CC fixation in distal clavicle fractures that are accompanied by CC disruption. We have not seen anchor pullout when used in associated with a clavicle plate for fracture indications. It is essential to recognize that the healing process of purely ligamentous disruption involving the AC and CC ligaments in AC joint dislocations differs from the healing observed in distal clavicle fractures with CC ligament disruption. Fixation of fractures promptly restores horizontal stability, reducing mechanical stress in the CC region, which explains differences in our observation in anchor pullout between these two clinical situations.

This study has several limitations. Firstly, being a retrospective study, it may be susceptible to bias. Second, the sample size is very small, with only three patients included in our report. The senior author stopped using this method of fixation for surgical treatment of AC joint dislocation after the third case and there were no additional cases. Third, all surgeries were performed by a single surgeon, potentially impacting the generalizability of our results. However, we believe that our findings contribute valuable insights to the existing literature on surgical treatment of ACJ dislocations, particularly regarding the use of suture anchors. This information can assist surgeons in selecting optimal techniques for addressing these complex injuries.

## Conclusion

In our case series, we found a high rate of fixation failure and early loss of reduction with the use of suture anchors for treatment of ACJ dislocation. These findings should be taken into consideration when selecting an appropriate implant for fixation of ACJ dislocation.

## Disclaimers:

Funding: This study is funded by 10.13039/100023503NYU Langone Health, no external funding or support was received for completion of this study.

Conflicts of interest: Mandeep S. Virk is a paid consultant for Exactech, Inc. All the other authors, their immediate families, and any research foundations with which they are affiliated have not received any financial payments or other benefits from any commercial entity related to the subject of this article.
